# Bee Bread as a Promising Source of Bioactive Molecules and Functional Properties: An Up-To-Date Review

**DOI:** 10.3390/antibiotics11020203

**Published:** 2022-02-05

**Authors:** Meryem Bakour, Hassan Laaroussi, Driss Ousaaid, Asmae El Ghouizi, Imane Es-Safi, Hamza Mechchate, Badiaa Lyoussi

**Affiliations:** 1 Laboratory of Natural Substances, Pharmacology, Environment, Modeling, Health and Quality of Life (SNAMOPEQ), Faculty of Sciences Dhar El Mahraz, University Sidi Mohamed Ben Abdallah, Fez 30000, Morocco; meryem.bakour@usmba.ac.ma (M.B.); hassan.laaroussi@usmba.ac.ma (H.L.); driss.ousaaid@usmba.ac.ma (D.O.); asmae.elghouizi@usmba.ac.ma (A.E.G.); lyoussi@gmail.com (B.L.); 2 Laboratory of Inorganic Chemistry, Department of Chemistry, University of Helsinki, 00014 Helsinki, Finland; Imane.essafi1@usmba.ac.ma

**Keywords:** bee bread, bioactive molecules, health benefits, natural antioxidant, natural antibiotics

## Abstract

Bee bread is a natural product obtained from the fermentation of bee pollen mixed with bee saliva and flower nectar inside the honeycomb cells of a hive. Bee bread is considered a functional product, having several nutritional virtues and various bioactive molecules with curative or preventive effects. This paper aims to review current knowledge regarding the chemical composition and medicinal properties of bee bread, evaluated in vitro and in vivo, and to highlight the benefits of the diet supplementation of bee bread for human health. Bee bread extracts (distilled water, ethanol, methanol, diethyl ether, and ethyl acetate) have been proven to have antioxidant, antifungal, antibacterial, and antitumoral activities, and they can also inhibit α-amylase and angiotensin I-converting enzyme in vitro. More than 300 compounds have been identified in bee bread from different countries around the world, such as free amino acids, sugars, fatty acids, minerals, organic acids, polyphenols, and vitamins. In vivo studies have revealed the efficiency of bee bread in relieving several pathological cases, such as hyperglycemia, hyperlipidemia, inflammation, and oxidative stress.

## 1. Introduction

Bee products are natural products that are either secreted by the body of bees through glands, i.e., venom, wax, and royal jelly, or collected and processed by the bees, i.e., nectar, pollen from flowers, and resin [1]. In recent years, research trends for bioactive molecules of natural origin have led to a proliferation of studies on bee products, including honey, propolis, royal jelly, bee bread, and bee pollen [2]. Bee bread is a unique bee product that is poorly known because, until a few years ago, beekeepers did not know the appropriate method for collecting this product without partially destroying the hive [3].

**Figure 1:**
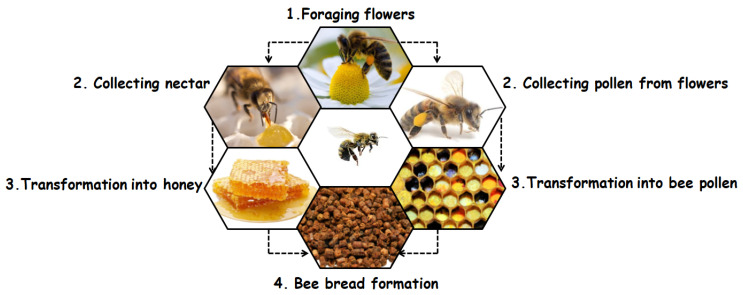
The process of forming bee bread in the hive.

For honey bees, the nutrients necessary for the survival and maintenance of the health of colony populations come from two main sources, namely nectar/honeydew and pollen from flowers. Nectar and honeydew provide carbohydrates and pollen provides other dietary needs, such as lipids, proteins, vitamins, and minerals [4]. However, bees do not directly consume nectar/honeydew or pollen; in both cases, they induce biochemical changes, such that the nectar/honeydew turns into honey and the pollen turns into bee bread [5]. The process of making bee bread begins with the collection of pollen from flowers, which is then mixed by bees with the nectar or honey and saliva containing digestive enzymes [6]. At this stage, the pollen from flowers is transformed into bee pollen, stored in the pollen basket in the bee’s hind legs, and carried to the hive, where the non-flying bees fill the cells of the hive with a mixture of bee pollen and honey. Then, a thin layer of wax is added to protect the mixture from oxygen. An anaerobic lactic fermentation process takes place, and the bee bread is produced (Figure 1). This type of lactic fermentation makes the bee bread more digestible and enriched with new nutrients [7]. Studies on the chemical composition of bee bread have shown that it is generally made up of water, protein, free amino acids, carbohydrates, fatty acids, and other bioactive molecules. This composition varies from one region to another depending on the honey plants’ climatic conditions and seasonal variations. All these conditions make bee bread a potential functional food with distinct bioactive molecules [8,9].

In the life of bees, there are times when they are highly active, such as when colonies are reproducing or during periods of high wax production. At these times, the demand for pollen rises, and a decrease in pollen inflow (fall and early spring) or the existence of pollen of poor nutritional quality will have serious consequences for the colony. This directs bees to rely on their bee bread store for their needs [10]. In this review, we will underline the importance of bee bread, not only for bee food and the good health of the hive, but also its nutritional importance for humans and its richness in bioactive molecules with protective or curative effects.

## 2. Methodology

A comprehensive literature search was performed from October 2021 to January 2022. The search engines “Google-Scholar, Web of Science, Scopus, Science-Direct and Pub-Med” were used to collect information on bee bread, its chemical composition, and its biological activities in vitro and in vivo using keywords such as “composition of bee bread”, “bee bread and patients”, the antimicrobial effect of bee bread”, “antiviral effect of bee bread”, “bee bread and rats”, and“effects of bee bread”. The papers were collected, examined for relevance, and then their general ideas were summarized.

## 3. Bee Bread Composition

### 3.1. Free Amino Acids

Several studies have investigated the free amino acids content of bee bread. For instance, Malaysian bee bread was analyzed in two studies published by Mohammad et al. [11] and by Othman et al. [12]. In the first study, four multifloral bee breads were analyzed using chromatographic separation; the botanical origins of the samples were *Mimosa pudica*, *Sphagneticola trilobata*, *Bidens pilosa*, *Cassia* sp, *Areca catechu*, *Peltophorum pterocarpum*, *Phaleria capitata*, *Cassia siamea*, *Citrus aurantifolia*, and *Ageratum conyzoides.* In the second study, three samples were analyzed using the WatersAccQ Tag method. The results of the two studies revealed the presence of the following amino acids in bee bread samples: phenylalanine, valine, histidine, methionine, isoleucine, leucine, threonine, alanine, arginine, tyrosine, glycine, proline, hydroxyproline, serine, glutamic acid, aspartic acid, and lysine. Moreover, Donkersley et al. [13] studied fifty-one samples of bee bread from England, whose botanical origins were *Trifolium, Impatiens, Rubus, Acer, Cirsium, Euscaphis, Cryptotaenia, Glycine*, *Coriandrum*, *Rosa, Prunus, Taraxacum, Camelina, Ranunculus, Salix*, and *Andira*. The ultra-high-performance liquid chromatography (UHPLC) analysis of these samples showed the presence of aspartate, glutamate, asparagine, serine, glutamine, histidine, glycine, threonine, arginine, alanine, γ-aminobutyric acid, tyrosine, cysteine, valine, methionine, tryptophan, phenylalanine, isoleucine, leucine, lysine, and proline. The gas chromatography-mass spectrometry (GC-MS) analysis of two American samples studied by DeGrandi-Hoffman et al. [14] showed the presence of the following amino acids: alanine, aspartic acid, glutamine, serine, leucine, isoleucine, methionine, threonine, valine, tryptophan, cysteine, phenylalanine, and proline. In addition, Bayram et al. [15] analyzed five Turkish bee bread samples, and they found the following amino acids: tryptophan, taurine, l-tyrosine, l-phenylalanine, l-isoleucine, l-leucine, gamma-aminobutyric acid, 3-amino isobutyric acid, l-methionine, l-2-aminoadipic acid, beta-alanine, l-aspartic acid, l-glutamic acid, l-valine, l-2-aminobutyric acid, ethanolamine, l-alanine, l-threonine, l-serine, l-glycine, l-asparagine, l-glutamine, l-proline, sarcosine, l-arginine, l-cystathionine, l-cystine, histidine, l-ornithine, l-carnosine,l- lysine, and l-anserine(Table 1).

The origin of the amino acids in bee bread is generally attributed to both floral (nectar, honeydew, and main pollen) and animal (bee secretions) origins. Since the main source is pollen, the amino acid profile or certain characteristics of the amino acids could be important for the botanical classification of some bee products, such as bee pollen, honey, and bee bread. For instance, tryptophan is considered a promising biomarker of acacia-related bee products, and arginine is a marker for chestnut-based hive products [16]. In addition to their role in the authentication of bee products, the amino acids contained in bee bread have been previously documented to have vital physiological roles in the human body. Accordingly, methionine, an essential amino acid, presents highly in functional foods, including bee products, participates in the DNA methylation reaction and protein synthesis, acts as glutathione (GSH) precursor, and acts as a powerful antioxidant agent by eliminating excess reactive oxygen species (ROS) and thus protects tissues from oxidative stress [17]. Arginine, an indispensable gluconeogenic amino acid, possesses immunomodulatory actions, including enhancing T cell (CD8^+^) and macrophage functions [18]. Therefore, the incorporation of bee bread into the daily human diet as a valuable nutraceutical food complement is needed.

### 3.2. Sugars

Bees use sugars to produce the energy necessary for their survival. Several studies have been carried out on the sugar content of bee bread. For instance, bee workers need about 4 mg/day of sugars to cover their daily energy [3,19]. The free sugar contents in bee bread were determined using high-performance liquid chromatography−refractive index detection (HPLC-RID) in a Moroccan multifloral sample (*Bupleurum spinosum*, *Anethum graveolens*, *Calendula officinalis*, *Anacyclus*, *Quercus ilex*, *Eucalyptus*, *Punica granatum*, and *Acacia*). The results showed that fructose was the major representative sugar with a concentration of 11.8 ± 0.6 g/100 g, followed by glucose (5.7 ± 0.4 g/100 g), and a small amount of trehalose (0.92 ± 0.01 g/100 g [20]). Similarly, Dranca et al. [21] documented that Romanian bee bread contains 19.73 g/100 g of fructose and 8.82 g/100 g of glucose, and a low percentage of melezitose and raffinose (0.97 g/100 g and 0.96 g/100 g, respectively). Moreover, the findings of Urcan et al. [22] for five multifloral bee bread samples (Brassicaceae, Poaceae, Myrtaceae, Rutaceae, Asteraceae, Fabaceae, Tiliaceae, Fabaceae, Rosaceae, Plantaginaceae, Fabaceae, Asteraceae, Lamiaceae, Salicaceae, Rosaceae, and Fagaceae) from Romania and India revealed by high-performance liquid chromatography diode array detection (HPLC-DAD) analysis showed that fructose was the major individual sugar, with values ranging from 13.97 ± 0.05 g/100 g to 19.58 ± 0.03 g/100 g, followed by glucose (6.40 ± 0.010 g/100 g to 15.13 ± 0.02 g/100 g), and smaller quantities of maltose and turanose(0.82 ± 0.02 g/100 g to 1.00 ± 0.01 g/100 g and 0.56 ± 0.02 g/100 g to 0.87 ± 0.01 g/100 g, respectively).

On the contrary, in a study published by Mohammad et al. [11], a sugar profile revealed that glucose was the major free sugar contained in four Malaysian multifloral bee breads analyzed by HPLC coupled with an evaporative light scattering detector (ELSD); the values ranged between 10.270 ± 0.140 g/100 g and 12.397 ± 0.980 g/100 g.The second highest sugar content was sucrose, ranging from 0.595 ± 0.000 g/100 g to 2.094 ± 0.141 g/100 g; then fructose, ranging from 0.396 ± 0.000 to 1.488 ± 0.140 g/100 g; and maltose, ranging from 0.694 ± 0.140 to 1.994 ± 0.000 g/100 g) (Table 1). This high variability could be explained by numerous factors, such as the harvesting time, the botanical origin, and the decomposition of sugars by lactic acid bacteria [23].

### 3.3. Fatty Acids

Fatty acids are among the most important compounds in bee bread. Bakour et al. [20] analyzed Moroccan bee bread using gas chromatography coupled with flame ionization detection (GC-FID), and they identified and quantified fourteen saturated and eleven unsaturated fatty acids, in which the highest levels were represented by α-linolenic and arachidonic acid, 25 ± 1% and 23.2 ± 0.5%, respectively. On the other hand, Turkish bee bread was analyzed by Kaplan et al. [24,25] in two studies using GC-MS analysis and GC-FID analysis, and the results indicated that seventeen saturated fatty acids and twenty unsaturated fatty acids were detected in eight monofloral samples in the first study, and five monofloral samples in the second study. In a recent study published by Drancaet al. [21], the GC-MS analysis of Romanian bee bread showed a content of thirty-seven fatty acids, with a percentage of 23.13% for saturated fatty acids and 76.87% unsaturated fatty acids (Table 1). Keeping in view the previous investigations, it is well recognized that individual fatty acids are the same in different bee breads; however, their concentrations vary depending on the geo-climatic conditions, the floral origin of pollen flowers, or even within the same species of the same growth site [26,27].

It has been documented that fatty acids play a crucial role in the nutrition, reproduction, and development of bees [28]. Owing to their antibiotic functions, such as antibacterial activity and antifungal activity, fatty acids, especially lauric, myristic, linoleic, and linolenic acids, contribute greatly to bee colony hygiene [29].

### 3.4. Minerals

The minerals from soil are carried into plants by their roots, and into bee bread through the pollen of flowers, nectar, or honeydew. Bee bread is one of the richest hive products in terms of macro and microelements. Moroccan bee bread was analyzed by Bakour et al. [20],who showed that potassium (338 ± 8 mg/100 g) was the predominant mineral followed by phosphorus (251 ± 4 mg/100 g), calcium (198 ± 4 mg/100 g), magnesium (61 ± 2 mg/100 g), iron (27.3 ± 0.3 mg/100 g), sodium (14.2 ± 0.1 mg/100 g), zinc (3.31 ± 0.04 mg/100 g), and manganese (2.6 ± 0.1 mg/100 g). The mineral content of bee bread may vary depending upon the agro-climatic conditions, specific melliferous plants, growth site, and harvesting season. Moreover, the methods used for sample collection and conditioning storage could affect the concentration and the mineral composition of bee bread [11].

In Malaysian bee bread, the most abundant mineral was potassium (6524.9 ± 610.6 mg/kg), followed by phosphorus (6402.28 ± 163.29 mg/kg), and magnesium (1635.4 ± 215.4 mg/kg) [11]. Similarly, Eleazu et al. [30] reported that calcium, phosphorus, potassium, magnesium, iron, zinc, and manganese were detected at high concentrations in Malaysian bee bread. In addition, the ICP-MS analysis of Serbian bee bread samples showed their richness in sodium, magnesium, potassium, calcium, manganese, selenium, zinc, and copper. The most abundant minerals were potassium, calcium, and magnesium [31] (Table 1).

Mineral elements are involved in the functioning of many biochemical and physiological processes in humans. For instance, a diet rich in potassium, an oligoelement that presents highly in bee bread samples of different floral and geographical origins, improves blood pressure and prevents cardiovascular diseases in both human and animal models [32,33]. Phosphorus, the sixth most abundant metal in the human body, is closely related to nucleic acid synthesis, enzyme activity, skeletal and non-skeletal muscle function, bone mineralization, energy, and lipid metabolism [34].

Micro-elements, including antioxidant minerals, such as zinc, iron, manganese, selenium, and copper, act as cofactors of many antioxidant and non-antioxidant enzymes. Therefore, they are effective components of metabolism and human body integrity [35].

### 3.5. Organic Acids

Organic acids are responsible for the microbial and digestive properties of bee products, and they are also used as food preservatives [36]. The information available in the literature on the organic acid content of bee bread remains limited. In a recent study conducted by Dranca et al. [21], the HPLC-DAD analysis of Romanian bee bread revealed the presence of the following organic acids: gluconic acid (79.2 g/kg), formic acid (6.75 g/kg), acetic acid (10.7 g/kg), propionic acid (1.3 g/kg), and butyric acid (0.33 g/kg). While in the Moroccan bee bread studied by Bakour et al. [20], the only organic acid found was oxalic acid. Organic acid content in bee bread varies depending upon the botanical origin (age, species, and vegetable tissue) (Table 1). Since bee bread is a bee pollen-derived product, its organic acid content can be compared to that of bee pollen. Kalaycioglu and coworkers [36] analyzed six multifloral and four monofloral bee pollen samples collected from different regions of Turkey and reported that gluconic acid (5.9–32 g/kg), lactic acid (0.72–1.2 g/kg), tartaric acid (0.17–0.30 g/kg), succinic acid (0.092–0.40 g/kg), and citric acid (0.19–0.31 g/kg) were the most quantified organic acids. Gluconic acid, a main organic acid in bee bread, exhibited potent antibacterial activities against anaerobic *(Porphyromonasgingivalis*), Gram-negative (*Escherichia coli*), and Gram-positive bacteria (*Staphylococcus aureus*), as well as achieved effective biofilm penetration. Importantly, polymyxin B associated with gluconic acid exhibited good biofilm penetration and high antibacterial action [37]. In addition, acetic acid, another valuable component found in bee products, has been documented to have anti-fungal effects against toxigenic *Aspergillus flavus* [38]. Therefore, bee bread extracts could be used as promising antibiotic and natural food preservative agents.

### 3.6. Vitamins

Vitamins are organic compounds tha thave several biochemical roles in growth, the regulation of mineral metabolism, and cell differentiation; some also possess antioxidant activity and others play the role of precursors of enzyme cofactors [39]. According to the literature, vitamin content is highly dependent on the plant origin of bee bread. Loper et al. [40] conducted a comparative study of the vitamin content in bee pollen and bee bread samples after seven, twenty-one, and forty-two days from the same plant source (*Prunus dulcis*), and they found that the vitamin content decreased by passing pollen from the flowers throughout the forty-two days of storage. Until present, few studies (Table 1) have been conducted on the vitamin composition of bee bread. Bakour and coworkers [20] reported that Moroccan bee bread contained α-tocopherol and δ-tocopherol with concentrations of 10.5 ± 0.8 and 0.4 ± 0.04 mg/100 g of bee bread (BB), respectively. Vitamin quantification was also the subject of other investigations. In fact, Salma Malihah et al. and Chinedum et al. [11,20,31] studied Malaysian bee bread and revealed the presence of vitamin C (11.52 mg/100 g), α-tocopherol, δ-tocopherol, vitamin A (146.8 mg/100 g), vitamin E (46.27 mg/100 g), thiamine (6.20 mg/100 g), and riboflavin (1.50 mg/100 g) (Table 1).

Since there is no specification for the vitamin content in bee bread, a bee pollen-derived product, the specifications for the vitamin composition of bee pollen suggest contents between 0.6 and 2 mg/100 g for riboflavin and between 0.6 and 1.3 mg/100 g for thiamine [41], thus allowing us to highlight bee bread as a dietary supplement and natural source of valuable vitamins.

### 3.7. Polyphenolic Composition

Owing to the development of advanced techniques for the separation and purification of molecules, such as gas chromatography (GC) and high-performance liquid chromatography (HPLC), as well as other identification techniques, such as mass spectroscopy (MS), thin-layer chromatography (TLC), and other combined techniques, several bioactive components have been identified in bee bread from different geographical origins (Table 1).

Polyphenol compounds are secondary metabolites produced by plants in response to different biotic and abiotic stress conditions [42]. These chemical compounds are divided into two groups: hydrophilic antioxidants, such as vitamin C and phenolic acids, and lipophilic antioxidants, such as carotenoids, tocopherols, and flavonoids [43]. The high content of antioxidants in bee bread is responsible for its bioactivities. For instance, Oltica and coworkers [44] found that there was a good correlation between the content of antioxidants (polyphenols, flavonols, flavones, and flavanones) and the antioxidant activity of scavenging free radicals carried out by 2,2-diphenyl-1-picrylhydrazyl (DPPH), trolox equivalent antioxidant capacity (TEAC), and ferric reducing antioxidant power (FRAP) tests, which explains their contribution to the antioxidant effects of bee bread extracts. It is well known that the soil nature, geographical conditions, and plant species highly influence the phytochemical composition of functional foods, including bee product extracts [7]. Furthermore, the solvent and extraction method used influence the quantity and the selectivity of the extract components [45]. Chemical variability has been observed in the composition of bee bread extracts of different geographical and floral origins.

The phenolic screening of multifloral Moroccan bee bread by HPLC showed the presence of thirteen phenolic compounds, where isorhamnetin-*O*-hexosyl-*O*-rutinoside was the most representative [20]. Similarly, Sobral et al. [46] analyzed six bee bread samples harvested from different apiaries located in the north of Portugal. High-performance liquid chromatography–diode array detection coupled to tandem mass spectrometry (HPLC-DAD-ESI/MS) analysis revealed the presence of thirty-two phenolic compounds, of which flavonol derivatives, mainly quercetin, kaempferol, myricetin, isorhamnetin, and herbacetin glycoside derivatives, were the main quantified antioxidant molecules.

A study conducted by Urcan et al. [22] revealed that five samples of bee bread from Romania and India contained kaempferol-3-*O*-glycoside, a hydroxycinnamic acid derivative, quercetin-3-*O*-sophoroside, a kaempferol-3-*O*-derivative, a hydroxycinnamic acid derivative, myrcetin, trycetin, luteolin, herbacetin-3-*O*-glycoside, quercetin-3-*O*-glycoside, and isoramnethin-3-*O*-glycoside. Similarly, Lithuanian, Romanian, Turkish, Portuguese, and Georgian bee bread samples showed the presence of several polyphenolic components belonging to different chemical groups, such as p-coumaric acid, kaempferol, chrysin, apigenin, caffeic acid, rosmarinic acid, myricetin, luteolin, naringin, rutin, and quercetin [15,21,47,48,49]. In addition, the GC-MS analysis of three bee bread samples from Poland showed the presence of kaempferol and apigenin [50] (Table 1).

Flavonoids, phenolic acids, and other antioxidant components are recognized as effective functional molecules, displaying numerous bio-pharmacological potentialities. In fact, several researchers suggest the use of bee bread as a common food preservative and bio-functional dietary supplement, or as a therapeutic strategy in the prevention of oxidative stress and associated ailments, such as cardio-metabolic, neurodegenerative, and cancerous diseases [46,51,52].

### 3.8. Microorganisms

Bee bread is a complex product that contains different microorganisms, such as bacteria and fungi, involved in the production process of bee bread to enrich it with newly produced nutrients. The micro-organisms found in bee bread originate from the bee’s saliva, added to pollen as a raw matter of bee bread. This microbiome plays an important role in the sustenance of larva and in adult bee’s health, including in making carbohydrates digestible and providing different nutrients with added value. Furthermore, the richness of bee bread in microorganisms can give it the properties of a probiotic product [53]. A study conducted by Dimov et al. [54] showed the following fungi composition in bee bread: *Cladosporium, Penicillium, Alternaria, Monilinia, Sclerotinia, Ascosphaera, Toxicocladosporium, Botrytis, Pseudopithomyces, Camerosporium, Paraconiothyrium, Podosphaera, Golovinomyces, Paraphaeosphaeria, Periconia*, and *Septoriella*. In addition, it was found that bee bread was rich in lactic acid bacteria, such as *Apilactobacilluskunkeei*, *Lactiplantibacillus plantarum*, *Fructobacillusfructosus*, *Levilactobacillus brevis*, *Lactobacillus delbrueckii*, *Lactobacillus musae*, *Lactobacillus crustorum*, and *Lactobacillus delbrueckii* [54,55,56].

### 3.9. Enzymes

Bee bread contains high amounts of enzymes, of which amylase, invertase, phosphatases, transferases, and glucose oxidase are the most important. Invertase and glucose oxidase are mainly produced in the hypopharyngeal glands, and are added by the bees to complete the honey maturation process. However, some enzymes, such as catalase and phosphatase, originate from nectar, honeydew, or pollen [57]. Enzyme cofactors, such as biotin, glutathione, and NAD, have also been found in bee bread. These enzymes can transform high molecular weight compounds into other low molecular weight molecules, such as polysaccharides and proteins. This makes bee bread more digestible than bee pollen [58,59].

**Table 1:** The chemical composition of bee bread from different countries in the world.

Component		Method Used	Country	Botanical Origin	References
Free amino acids	Tryptophan, Taurine, l-Tyrosine, l-Phenylalanine, l-isoleucine, l-Leucine, Gamma-aminobutyric acid, 3-Amino isobutyric acid, l-Methionine, l-2-Aminoadipic acid, Beta-Alanine, l-Aspartic acid, l-Glutamic acid, l-Valine, l-2-aminobutyric acid, Ethanolamine, l-Alanine, l-Threonine, l-Serine, l-Glycine, l-Asparagine, l-Glutamine, l-Proline, Sarcosine, l-Arginine, l-Cystathionine, l-Cystine, Histidine, l-ornithine, l-Carnosine, l-Lysine, and l-Anserine	LC-MS/MS	Turkey	Five samples (not determined)	[15]
	Phenylalanine, Valine, Histidine, Methionine, Isoleucine, Leucine, Threonine, Alanine, Arginine, Tyrosine, Glycine, Proline, Hydroxyproline, Serine, Glutamic acid, Aspartic acid, andLysine	Chromatographic separation	Malaysia	Four multifloral samples (*Mimosa pudica, Sphagneticolatrilobata, Bidens ilosa, Cassia* sp, *Areca catechu, Peltophorumpterocarpum, Phaleria capitata, Cassia siamea, Citrus aurantifolia,* and *Ageratum conyzoides*)	[11]
	Aspartate, Glutamate, Asparagine, Serine, Glutamine, Histidine, Glycine, Threonine, Arginine, Alanine, γ-aminobutyric acid, Tyrosine, Cysteine, Valine, Methionine, Tryptophan, Phenylalanine, Isoleucine, Leucine, Lysine, and Proline	UHPLC	England	Fifty-one samples of bee bread (*Trifolium, Impatiens, Rubus, Acer, Cirsium, Euscaphis, Cryptotaenia, Glycine, Coriandrum, Rosa, Prunus, Taraxacum, Camelina, Ranunculus, Salix, and Andira*)	[13]
	Alanine, Aspartic acid, Glutamine, Serine, Leucine, Iso-leucine, Methionine, Threonine, Valine, Tryptophan, Cysteine, Phenylalanine, and Proline.	(GC/MS)	USA	Two samples (not determined)	[14]
	Alanine, Arginine, Aspartic acid, Glutamic acid, Glycine, Histidine, Hydroxyproline, Isoleucine, Leucine, Lysine, Methionine, Phenylalanine, Proline, Serine, Threonine, Tyrosine, and Valine	Waters AccQ Tag method	Malaysia	Three samples (not determined).	[12]
Sugars	Trehalose, Glucose, and Fructose	HPLC-RID	Morocco	One multifloral sample *(Bupleurum spinosum, Anethum graveolens, Calendula officinalis, Anacyclus, Quercus ilex, Eucalyptus, Punica granatum,* and *Acacia)*	[20]
	Fructose, Glucose, Melezitose, andRaffinose	HPLC-RID	Romania	One sample (not determined).	[21]
	Fructose, Glucose, Turanose, and Maltose	HPLC-DAD	Romania and India	Five samples (Brassicaceae, Poaceae, Myrtaceae, Rutaceae, Asteraceae, Fabaceae, Tiliaceae, Fabaceae, Rosaceae, Plantaginaceae, Fabaceae, Asteraceae, Lamiaceae, Salicaceae, Rosaceae, and Fagaceae)	[22]
	Fructose, Glucose, Sucrose, and Maltose	HPLC coupled with an ELSD	Malaysia	Four multifloral samples (*Mimosa pudica, Sphagneticolatrilobata, Bidens ilosa, Cassia* sp, *Areca catechu, Peltophorumpterocarpum, Phaleria capitata, Cassia siamea, Citrus aurantifolia,* and *Ageratum conyzoides*)	[11]
Microorganisms	*Lactobacillus kunkeei*	RAPD-PCR	Turkey	Four samples (not identified).	[60]
	*Cladosporium, Penicillium, Alternaria, Monilinia, Sclerotina, Ascosphaera, Toxicocladosporium, Botrytis, Pseudopithomyces, Camerosporium, Paraconiothyrium, Podosphaera, Golovinomyces, Paraphaeosphaeria, Periconia,* and *Septoriella*	NGS-sequencing	Bulgaria	Four samples (not identified).	[54]
	*Apilactobacilluskunkeei, Lactiplantibacillus plantarum, Fructobacillusfructosus, Levilactobacillus brevis,* and *Lactobacillus delbrueckii*	PCR-DGGE analysis RAPD-PCR analysis	Italy	Twelve samples (not identified)	[55]
Organic acids	Oxalic acid	UFLC-PDA	Morocco	One multifloral sample *(Bupleurum spinosum, Anethum graveolens, Calendula officinalis, Anacyclus, Quercus ilex, Eucalyptus, Punica granatum,* and *Acacia)*	[20]
	Gluconic acid, Formic acid, Lactic acid, Acetic acid, Succinic acid, Propionic acid, Butyric acid	HPLC-DAD	Romania	One sample (not determined)	[21]
Vitamins	Vitamin A, Vitamin E, Thiamine, andRiboflavin	HPLC	Malaysia	One sample (not determined)	[30]
	Vitamin C	Method AOAC 967.21	Malaysia	Four multifloral samples (*Mimosa pudica, Sphagneticolatrilobata, Bidens pilosa* *, Cassia* sp, *Areca catechu, Peltophorumpterocarpum, Phaleria capitata, Cassia siamea, Citrus aurantifolia,* and *Ageratum conyzoides*)	[11]
	α-Tocopheroland δ-Tocopherol.	HPLC	Morocco	One multifloral sample *(Bupleurum spinosum, Anethum graveolens, Calendula officinalis, Anacyclus, Quercus ilex, Eucalyptus, Punica granatum, and Acacia)*	[20]
	Octanoic, Decanoic, Undecanoic, Dodecanoic, Tridecanoic, Tetradecanoic, Pentadecanoic, Hexadecanoic, Palmitoleic, Heptadecanoic, Stearic, Oleic, Linoleic, α-Linolenic, Arachidic, Gadoleic, Eicosadienoic, Heneicosanoic, Eicosatrienoic, Arachidonic, Eicosatrienoic, Behenic, Eicosapentaenoic, Lignocerin, andNervonicacid.	GC-FID	Morocco		[20]
Fatty acids	Butanoic, Hexanoic, Octanoic, Decanoic, Undecanoic, Dodecanoic, Tridecanoic, Tetradecanoic, Pentadecanoic, Hexadecanoic, Heptadecanoic, Octadecanoic, Icosanoic, Heneicosanoic, Docosanoic, Tricosanoic, Tetracosanoic, (Z)-tetradec-9-enoic, (Z)-pentadec-10-enoic, (9Z)-hexadec-9-enoic, cis-10-heptadecenoic, (E)-octadec-9-enoic, (Z)-octadec-9-enoic, (Z)-icos-11-enoic, (Z)-docos-13-enoic, (Z)-tetracos-15-enoic, octadeca-9,12-dienoic, (9Z,12Z)-octadeca-9,12-dienoic, Octadeca-6,9,12-trienoic, Icosa-11,14-dienoic, (11Z,14Z,17Z)-icosa-11,14,17-trienoic, (5Z,8Z,11Z,14Z)-icosa-5,8,11,14-tetraenoic, Docosa-13,16-dienoic, (9Z,12Z,15Z)-octadeca-9,12,15-trienoic, (11Z,14Z,17Z)-icosa-11,14,17-trienoic, (5Z,8Z,11Z,14Z,17Z)-icosa-5,8,11,14,17-pentaenoic, and Docosa-4,7,10,13,16,19-hexaenoic	GC-MS	Turkey	Eight monofloral samples (*Trifolium pratense, Gossypium hirsutum, Castanea sativa, Citrus* spp., *Helianthus annuus,* and *Trifolium repens*)	[25]
	Butanoic acid, Hexanoic acid, Octanoic acid, Decanoic acid, Undecanoic acid, Dodecanoic acid, Tridecanoic acid, Tetradecanoic acid, (cis-9) (Z)-tetradec-9-enoic acid, Pentadecanoic acid, (cis-10) (Z)-pentadec-10-enoic, Hexadecanoic acid, (9Z)-hexadec-9-enoic acid, Heptadecanoic acid, cis-10-heptadecenoic, Octadecanoic acid, (E)-octadec-9-enoic, (Z)-octadec-9-enoic, (all-trans-9,12) Octadeca-9,12-dienoic acid, (all-cis-9,12) (9Z,12Z)-octadeca-9,12-dienoic acid, (all-cis-6,9,12) Octadeca-6,9,12-trienoic acid, (all-cis-9,12,15) Octadeca-6,9,15-trienoic acid, Icosanoic acid, (cis-11) (Z)-icos-11-enoic acid, (all-cis-11,14,) Icosa-11,14-dienoic acid, (all-cis-8,11,14) Icosa-8,11,14-trienoic acid, (all-cis-11,14,17) Icosa-11,14,17-trienoic acid, (all-cis-5,8,11,14) Icosa-5,8,11,14-tetraenoic acid, (all-cis-5,8,11,14,17) Icosa-5,8,11,14,17-pentaenoic, Heneicosanoic acid, Docosanoic acid, (cis-13) (Z)-docos-13-enoic, (all-cis-13,16) Docosa-13,16-dienoic, (all-cis-4,7,10,13,16,19) Docosa-4,7,10,13,16,19-hexaenoic, Tricosanoic acid, Tetracosanoic acid, and (cis-15) (Z)-tetracos-15-enoic	GC-MS	Romania	One sample (not determined)	[21]
	Butyric acid, Caproic acid, Caprylic acid, Capric acid, Undecanoic acid, Lauric acid, Tridecanoic acid, Myristic acid, Pentadecanoic, Palmitic acid, Heptadecanoic acid, Stearic acid, Arachidic acid, Heneicosanoic, Behenic acid, Tricosanoic acid, Lignoceric acid, Myristoleic acid, cis-Pentadecanoic acid, Palmitoleic acid, cis-Heptadecanoic acid, Elaidic acid, Oleic acid, cis-Eicosenoic, Erucic acid, Nervonic acid, Linolelaidic acid, Linoleic acid, g-Linolenic acid, cis-11,14-Eicosadienoic, cis-8,11,14-Eicosatrienoic acid, Arachidonic acid, cis-13,16 Docosadienoic, a-Linolenic acid, cis-11,14,17-Eicosatrienoic acid, cis-5,8,11,14,17-Eicosapentaenoic acid, and Docosahexaenoic acid	GC-FID	Turkey	In five monofloral samples, the majority of pollen is *Citrus* spp. Secondary pollens belonging to Asteraceae, Fabaceae, and Brassicaceae families	[24]
Minerals	Ca, Fe, K, Mg, Na, Zn, P, Pb, Ni, Se, Mn, Co, Cu, and Cd	ICP-AES	Morocco	One multifloral sample (*Bupleurum spinosum, Anethum graveolens, Calendula officinalis, Anacyclus, Quercus ilex, Eucalyptus, Punica granatum,* and *Acacia*)	[20]
	Ca, Fe, K, Mg, Mn, Na, Zn, P, and Se.	ICP-MS	Malaysia	Four multifloral samples (*Mimosa pudica, Sphagneticolatrilobata, Bidens ilosa, Cassia* sp, *Areca catechu, Peltophorumpterocarpum, Phaleria capitata, Cassia siamea, Citrus aurantifolia,* and *Ageratum conyzoides*	[11]
	Na, Mg, K, Ca, Mn, Se, Fe, Zn, and Cu.	ICP-MS	Serbia	Twelve bee bread samples (not determined)	[31]
	Ca, P, K, Mg, Fe, Zn, and Mn	AAS	Malaysia	One sample (not determined)	[30]
Polyphenols composition	Kaempferol-3-*O*-glycoside, Hydroxycinnamic acid derivative, Quercetin-3-*O*-sophoroside, Kaempferol-3-*O*-derivative, Hydroxycinnamic acid derivative, Myricetin, Trycetin, Luteolin, Herbatin-3-*O*-glycoside, Quercetin-3-*O*-glycoside, and Isoramnethin-3-*O*-glycoside	HPLC-DAD	Romania and India	Five samples (Brassicaceae, Poaceae, Myrtaceae, Rutaceae, Asteraceae, Fabaceae, Tiliaceae, Fabaceae, Rosaceae, Plantaginaceae, Fabaceae, Asteraceae, Lamiaceae, Salicaceae, Rosaceae, and Fagaceae)	[22]
	*p*-Coumaric acid, Kaempferol, Chrysin, and Apigenin	HPLC	Lithuania	Nine simples (not determined)	[48]
	Caffeic acid, p-Coumaric acid, Rosmarinic acid, Myricetin, Luteolin, Quercetin, and Kaempferol	HPLC-DAD	Romania	One sample (not determined)	[21]
	Naringin, Rutin, and Quercetin.	HPLC	Georgia	Two samples	[49]
	Kaempferol and Apigenin	GC/MS	Poland	Three samples.	[50]
	Kaempferol-*O*-hexosyl-*O*-rutinoside, Quercetin-*O*-hexosyl-*O*-hexoside, Quercetin-*O*-hexosyl-*O*-hexoside, Methylherbacetrin-*O*-dihexoside, Isorhamnetin-*O*-hexosyl-*O*-rutinoside, Quercetin-*O*-pentosyl-hexoside, Quercetin 3-*O*-rutinoside, Methylherbacetrin-3-*O*-rutinoside, Isorhamnetin-*O*-pentosyl-hexoside, Isorhamnetin-*O*-pentosyl-hexoside, Kaempferol-3-*O*-rutinoside, Isorhamnetin-*O*-rhamnoside-hexoside, and Isorhamnetin-3-*O*-rutinoside	LC-DAD–ESI/MSn	Morocco	One multifloral sample (*Bupleurum spinosum, Anethum graveolens, Calendula officinalis, Anacyclus, Quercus ilex, Eucalyptus, Punica granatum*, and *Acacia*)	[20]
	Hesperetin, Quercetin-*O*-hexosyl-*O*-rutinosid, Quercetin-diglucoside, Methyl herbacetin-*O*-dihexoside, Kaempferol-*O*-dihexoside, Methyl herbacetin-*O*-rutinoside, Isorhamnetin-*O*-pentosyl-hexosid, Kaempferol-3-*O*-rutinoside, Quercetin-3-*O*-glucoside, Quercetin-*O*-malonyl hexoside, Kaempferol-*O*-malonyl hexoside, Di-p-coumaroylspermidine, Kaempferol-3-*O*-rhamnoside, and Isorhamnetin-*O*-deoxyhexoside	LC/DAD/ESI-MS	Portugal	Three samples. Sample 1: *Plantago* spp. (47%) Sample 2: *Crepiscapillaris* (60%) Sample 3: *Cytisus striatus* (48%)	[7]
	2,5-Dihydroxybenzoic acid, 2-Hydroxycinnamic acid, Caffeic acid, Catechin, Epicatechin, Chlorogenic acid, Ethyl gallate, Gallic acid, Isorhamnetin, Kaempferol, Luteolin, Myricetin, Naringin, p-Coumaric acid, Phlorizin, Propyl gallate, Protocatechuic acid, Quercetin, Resveratrol, Rutin, Salicylic acid, Sinapic acid, Syringic acid, Trans ferulic acid	LC-MS/MS	Turkey	Five samples (not identified).	[15]
	Myricetin-3-*O*-rutinoside, Quercetin-*O*-hexosyl-*O*-rutinoside, Kaempfrol-*O*-hexosyl-*O*-rutinoside,Quercetin-*O*-hexosyl-*O*-hexoside, Isorhamnetin-*O*-hexosyl-*O*-rutinoside, Methyl herbacetrin-*O*-dihexoside, Myricetin-3-*O*-glucoside, Quercetin-*O*-pentosyl-hexoside, Quercetin-*O*-hexosyl-rutinoside, Quercetin 3-*O*-rutinoside, Methyl herbacetrin-*O*-hexosyl-rutinoside, Kaempferol-*O*-dihexoside, Methyl herbacetrin-3-*O*-rutinoside, Methyl herbacetrin-*O*-dihexoside, Kaempferol-*O*-hexosyl-rutinoside, Isorhamnetin-*O*-pentosyl-hexoside, Isorhamnetin-*O*-hexosyl-rutinoside, Kaempferol-3-*O*-rutinoside, Quercetin-3-*O*-glucoside, Isorhamnetin-o-pentosyl-hexoside, Isorhamnetin-*O*-pentosyl-hexoside, Acetyl kaempferol-*O*-deoxyhexosyl-hexoside, Methyl herbacetrin-3-*O*-glucoside, Laricitrin-3-*O*-rhamnoside, Isorhamnetin-3-*O*-rutinoside, Quercetin-3-*O*-rhamnoside, Kaempferol-o-pentosyl-deoxyhexoside, Isorhamnetin-3-*O*-glucoside, Acetyl kaempferol-*O*-hexoside, Kaempferol-3-*O*-rhamnoside, Isorhamnetin-3-*O*-rhamnoside, Acetyl isorhamnetin-*O*-hexoside	HPLC-DAD-ESI/MS	Portugal	Six samples (not identified)	[46]

## 4. The Bioactive Effect of Bee Bread: In Vitro Investigations

### 4.1. Antioxidant Capacity of Bee Bread

As already mentioned, antioxidant molecules are one of the most important constituents of bee bread. Several studies have evaluated the antioxidant capacity of these molecules using spectrophotometric techniques. Akhir et al. [61] and Othman et al. [12] tested the antioxidant capacities (DPPH, ABTS, and FRAP tests) of different Malaysian bee bread extracts (hexanoic, ethanolic, and distilled water extracts). The authors showed that the solvents used influenced the bioactivity of the bee bread. The most potent extract was the ethanolic extract, followed by the hexanoic extract and then the aqueous extract. Similarly, 15 Colombian bee bread samples were analyzed by Zuluaga et al. [62] for their antioxidant activity. The authors revealed that all the extracts had good antioxidant activity with FRAP and TEAC methods; their total flavonoid content ranged between 1.9 and 4.5 mg equivalent of quercetin/g, while their total phenolic content ranged between 2.5 and 13.7 mg equivalent of gallic acid/g.

The antioxidant activity of Moroccan bee bread was analyzed in three studies conducted by Bakour et al. [20,63,64] using three different solvents for extraction (ethanol, ethyl acetate, and methanol). The results showed that the antioxidant activity evaluated by three tests—DPPH, ABTS, and reducing power—had the lowest recorded values of IC_50_/EC_50__50_ forthe ethanolic extract, followed by the methanolic extract and then by the ethyl acetate extract.

Several studies published between 2004 and 2020 demonstrated the antioxidant capacity of European bee bread in vitro. The results showed that all evaluated samples had potent antioxidant activities [50,65,66,67,68] (Table 2).

### 4.2. The Antitumoral Effect of Bee Bread

The antitumor activity of bee bread has been evaluated in vitro in two studies, the first performed by Markiewicz-Żukowska et al. [50], in which different ethanolic extracts of bee bread samples collected from Poland were tested for the viability of the glioblastoma cell line (U87MG) after 24 h, 48 h, and 72 h. The results showed that bee bread ethanolic extract reduced the viability of cancer cells with percentages ranging from 49% to 66%. This inhibitory effect appeared mainly after the passage of 72 h of contact.

The second study is that of Sobral et al. [46],who evaluated the antitumor activity of bee bread from northeastern Portugal against different human tumor cell lines—NCI-H460 (non-cellular lung cancer), HepG2 (carcinoma hepatocellular), HeLa (cervical cancer), MCF-7 (breast adenocarcinoma)—and also against non-tumor liver cells (porcine liver cells, PLP2). The bee bread samples showed low to moderate cytotoxicity ranging from >400 to 68 µg/mL; however, none of the bee bread samples showed toxicity against normal cells (Table 2).

### 4.3. Hypotensive Effect

The hypotensive effect of bee bread was evaluated by Nagai et al. [69] using enzymatic hydrolysates from bee bread prepared by three proteases: pepsin, trypsin, and papain. The obtained hydrolysates showed inhibitory activities for the angiotensin I converting enzyme of 1.48 mg protein/mL obtained by the hydrolyzate of pepsin, 2.16 mg protein/mL obtained by the hydrolyzate of trypsin, and 5.41 mg protein/mL obtained by papain hydrolysate (Table 2).

### 4.4. The Inhibition Effect of Carbohydrate-Hydrolyzing Enzymes

The good management of diabetes necessitates the control of the approach to the first line of treatment, such as discovering new agents with a high inhibition potential for carbohydrate-hydrolyzing enzymes. In vitro studies have demonstrated the ability of bee bread methanolic extract to inhibit alpha-amylase with an IC_50_ % of 3.57 mg/mL [67]. The techniques of molecular docking, ultraviolet absorption, and fluorescence quenching tests have demonstrated that the bee bread contains functional fatty acids thatinteract with the amino acid residues of hydrolyzing enzymes through hydrogens bonds and vander Waals interactions [70]. Bee bread is a complex product with a high flavonoid content; flavonoids are known for their capacity to embed in the active site of the enzyme by bond and alkyl interactions, which are facilitated through the diverse methyl and hydroxyl groups that characterize the flavonoid’s structure [71]. The ability of bee bread to control metabolism disorders through the inhibition of carbohydrate-hydrolyzing enzymes has been confirmed by in vivo studies detailing the pharmacological effects of bee bread, as shown in the in vivo investigations section (Table 2).

**Table 2:** In vitro studies of bee bread from different countries in the world.

Country	Functional Effect	The Majority of Pollen Grains Identified in Bee Bread (BB)	Extraction Used	Concentration	Extraction Time	Results Obtained	References
Malaysia	Antioxidant	One sample, not identified	Hexane and 70% ethanol using Soxhlet apparatus	Sixty gramsof sample extracted with 300 mL of solvent	2 h	DPPH value of ethanolic extract (%): 93.60 ± 0.03 DPPH value of hexanic extract (%): 83.81 ± 0.05 ABTS value of ethanolic extract (%): 97.95 ± 0.01 ABTS value of hexanicextract (%): 71.23 ± 0.01 FRAP value of ethanolic extract (mM FE/g): 0.85 ± 0.01 FRAP value of hexanic extract (mM FE/g): 2.41 ± 0.02	[61]
Portugal	Antioxidant	Three samples Sample 1: *Plantago* sp. (47%) Sample 2: *Crepis capillaris* (60%) Sample 3: *Cytisus striatus* (48%)	EtOH/H_2_O (80:20, *v/v*)	Two grams of sample was extracted with 40 mL of EtOH/H_2_O (80:20, *v/v*)		Total phenolic content value ranging between 3.2 and 3.8 ± 0.1 mg GAE/g Total flavonoid content value ranging between 0.6 and 2.7 mg QE/g	[47]
Ukraine	Antioxidant	Five samples	Methanol/water solution (70%, *v/v*)	A total of 0.1 g of sample in 5 mL of methanol/water solution (70%, *v/v*)		Total phenolic content value ranging between 12.36 and 25.44 mg GAE/g Total flavonoid content ranging between 13.56 and 18.24 µg QE/g	[72]
Lithuania	Antioxidant	Four samples	Not mentioned	Not mentioned	Not mentioned	Total phenolic content value ranging between 306 and 394 mg GAE/100g DPPH radical scavenging abilities ranged between 85 and 93%	[73]
Colombia	Antioxidant	15 samples, not identified	Ethanol (96% *v/v*)	One gram of sample extracted with 30 mL of solvent	24 h	FRAP value ranging between 35.0 and 70.1 μmoltrolox/g TEAC value ranging between 46.1 and 76.3 μmoltrolox/g Total flavonoid content value ranging between 1.9 and 4.5 mg eq-quercetine/g Total phenolic content value ranging between 2.5 and 13.7 mg eq-gallic acid/g	[62]
Lithuania	Antioxidant	One sample (not identified)	Three types of extraction: Hot distilled water Distilled water Ethanol	Extract 1: 3 g of BB was extracted by boiling with 10 volumes of distilled water. Extract 2: 3 g of BB was extracted by shaking with 10 volumes of distilled water. Extract 3: 3 g of BB with 10 volumes of ethanol	Extract 1: 1h Extract 2: 1 day Extract 3: 1 day	Superoxide anion radical scavenging abilities ranged between 9.92% and 100% Hydroxyl radical scavenging abilities ranged between 9.75 and 100% DPPH radical scavenging abilities ranged between 10.5 l ± 0.2% and 98.7 ± l 0.2% Antioxidant abilities ranged between 0.018 l ± 0.002 and 1.352 ± l 0.036%	[68]
Morocco	Antioxidant	One sample (not identified)	Ethanolic extraction (70%)	One gram of BB macerated in 20 mL of ethanol (70%)	1 week	Polyphenol content was 14.88 ± 0.98 mg GAE/g, flavonoid content was 1.67 ± 0.12 mg QE/g, total antioxidant capacity was 143.78 ± 11.38 mg AAE/g, IC_50_ of DPPH was 0.05 ± 0.01 mg/mL, IC_50_ of ABTS was 0.08 ± 0.05 mg/mL, and reducing power was 0.05 ± 0.04 mg/mL	[63]
Morocco	Antioxidant	One multifloral sample (35% *Anethum graveolens*, 24% *Quercus ilex*, 16% *Eucalyptus*, and 25% other pollens)	Hydromethanolic extract	One gram of BB extracted twice with 30 mL of a mixture of methanol/water (80:20 *v/v*)	60 min	Total antioxidant capacity: 143 ± 22 DPPH: 0.98 ± 0.06 ABTS: 0.50 ± 0.04 Reducing power: 0.19 ± 0.03	[20]
Ukraine	Antioxidant	Five samples (not determined)	BB was extracted with ethanol	A total of 0.1 g of BB was extracted with 20 mL of ethanol (80%)	2 h	Total polyphenol content ranged between 12.36 ± 0.34 mg GAE/g and 25.44 ± 0.22 mg GAE/g Total flavonoid content ranged between 13.56 ± 0.04 μg QE/g and 15.35 ± 0.09 μg QE/g The best value of the DPPH test was 15.78 mg TEAC/g The value for reducing power was 250 mg TEAC/g	[66]
Lithuania	Antioxidant	Nine samples (not determined)	Extraction of phenolic compounds using distilled water, methanol, and diethyl ether	Fifty grams of BB was extracted with 250 mL of distilled water and 250 mL of methanol; the residue obtained was dissolved in 5 mL of distilled water and extracted with 5 mL of diethyl ether (three times)	Not determined	DPPH values ranged between 64.2 ± 1.8% and 93.9 ± 0.6% ABTS values ranged between 76.5 ± 0.2% and 94.8 ± 0.5%	[48]
Morocco	Antioxidant	One sample (not identified)	BB was extracted with ethyl acetate	-	Not determined	Polyphenols: 27.27 ± 0.38 mg EqGA/g Flavonoids: 5.29 ± 0.27 mg EqQ/g TAC: 65.44 ± 6.34 mg EqAA/g IC_50_ of ABTS:1.52 ± 0.021 mg/mL IC_50_ of DPPH:0.43 ± 0.02 mg/mL EC_50_ of RP:0.71 ± 0.05 mg/ml	[64]
Poland	Antioxidant	Three samples (not identified)	Ethanolic Extract	Twenty grams were extracted with 80 g of 95% (*v/v*) ethanol and re-extracted with 40.0 g of 95%(*v/v*) ethanol	12 h	Polyphenolic content: ranged between 33.43 ± 0.7 and 36.52 ± 0.6 mg GAE/g Antioxidant activity: ranged between 0.56 ± 0.06 and 1.11 ± 0.09 mmol/L	[50]
	Antitumoral					The cytotoxicity of BB was studied using a glioblastoma cell line (U87MG), the results indicated a time-dependent inhibitory effect on the viability of U87MG cells treated with BB. The cell viability was decreased to 49–66% after 72 h.	
Turkey	α-amylase inhibition	One sample; predominant pollen was *Trifolium pretense* (70.39%), important minor pollen (3–15%) presented by Cistaceae, Asteraceae, Rosaceae, and others (<3%)	BB was frozen, powdered, and then extracted with methanol	Two grams was mixed with 10 mL of methanol	48h under stirring	IC_50_ of α-amylase inhibition was 3.57 ± 0.01, better than acarbose (IC_50_ = 5.93 ± 0.01)	[67]
Lithuania	Angiotensin I-converting enzyme inhibition	One sample (not determined)	Preparation of enzymatic hydrolysates from bee bread by digestion with three kinds of enzymes (pepsin, trypsin, and papain)	10% (Pepsin hydrolysate) 10% (Trypsin hydrolysate), 4% (Papain hydrolysate)	Not determined	IC_50_ of angiotensin I-converting enzyme inhibition of pepsin hydrolysate: 1.48 mg protein/mL IC_50_ of angiotensin I-converting enzyme inhibition of trypsin hydrolysate: 2.16 mg protein/mL IC_50_ of angiotensin I-converting enzyme inhibition of papain hydrolysate: 5.41 mg protein/mL	[69]
Portugal	Antitumor activity	Six samples, not determined	BB was lyophilized and extracted twice with methanol:water (80:20, *v/v*)	One gram of BB in 30 mL of solvent	1h	Three samples of BB hadcytotoxic activity against MCF-7 (breast adenocarcinoma), IG_50_ values were: 186 ± 6 µg/mL; 84 ± 3 µg/mL; and 164 ± 4 µg/mL. Three samples of BB had cytotoxic activity against NCI-H460 (non-small cell lung cancer), IG_50_ values were:253 ± 10 µg/mL; 85 ± 5 µg/mL; and 68 ± 8 µg/mL. Four samples of BB hadcytotoxic activity against HeLa (cervical carcinoma), IG_50_ values were:345 ± 13 µg/mL; 225 ± 12 µg/mL; 209 ± 21 µg/mL; and 366 ± 7 µg/mL. One sample of BB had cytotoxic activity against HepG2 (hepatocellular carcinoma), IG_50_ value was 67 ± 1 µg/mL. None of the BB samples had toxicity against non-tumor liver cells (porcine liver cells, PLP2).	[46]
Turkey	Antioxidant	One sample; predominant pollen was *Trifolium pretense* (70.39%), important minor pollen (3–15%) presented by Cistaceae, Asteraceae, Rosaceae, and others (<3%)	BB was frozen, powdered, and then extracted with methanol	Two grams was mixed with 10 mL of methanol	48 h under stirring	Total phenolics:6.93 ± 0.09 mg GAE/g; Total flavonoids:2.27 ± 0.05 mg QE/g; Antioxidant capacity:83.62 ± 0.33 μmol FeSO_4_ 7 H_2_O/g sample	[67]
Malaysia	Antioxidant	Three samples, not identified	Distilled water or ethanol 70%	Fifty grams of bee bread extracted with 10 volumes of distilled water or 70% of ethanol	72 h	DPPH value of aqueous extract ranging between 2.86% and 8.95% DPPH value of ethanolic extract ranging between 72.04% and 79.34% FRAP value of aqueous extract ranging between 0.94 mmol Fe^2^/L and 1 mmol Fe^2^/L FRAP value of ethanolic extract ranging between 1.07 mmol Fe^2^/L and 1.08 mmol Fe^2^/L Total flavonoid content of aqueous extract ranging between 2.88 mg QE/g and 3.92 mg QE/g Total flavonoid content of ethanolic extract ranging between 16.48 mg QE/g and 26.57 mg QE/g Total phenolic content of aqueous extract ranging between 14.19 mg GAE/g and 15.38 mg GAE/g Total phenolic content of ethanolic extract ranging between 21.32 mg GAE/g and 22.54 mg GAE/g	[12]

### 4.5. Probiotic Properties of Bee Bread

Probiotics are defined by the Food and Agriculture Organization and the World Health Organization as “live microorganisms that when administered in adequate amounts confer a health benefit on the host” [74]. Human probiotic strains frequently belong to the Lactobacilli, Bifidobacteria lactococcus, Streptococcus, and Enterococcusgenera, and some yeast strains belonging to the genus Saccharomyces [75]. Recently, the field of study on probiotics has gained great scientific and public interest due to their beneficial effects in the treatment of several human diseases, notably inflammatory bowel diseases, gut infections, allergy, asthma pulmonary infections, and even psychiatric illnesses [76,77]. A recent study suggested that probiotics could be beneficial in the framework of COVID-19 infection through their immunomodulatory effect [78]. Indeed, the relationship between probiotic bacteria and human well-being has been well established and, as a result, consumers are becoming more concerned and require more functional food in their diet, especially fermented food and beverages [79].

In a similar context, bee bread is a natural probiotic-rich product that contains a different and complex spectrum of microorganisms, such as bacteria and fungi, involved in the bee bread production process through bee pollen lactic fermentation [80]. Indeed, Toutiaee et al. [81] reported the isolation of a Bacillus species with probiotic properties.

The microbial composition with probiotic properties of bee bread confers interesting therapeutic effects to this product. Khalifa et al. [82] in their review study suggested that bee bread exhibits a remarkable hypolipidemic effect through the cholesterol-lowering factors produced by Lactobacillus bacteria. Moreover, the presence of Lactobacillus bacteria and fructophilic lactic acid bacteria in bee bread provides a promising source of compounds with high techno-functional properties that are used in the food industry as a food preservative or in functional culture for the production of fermented food at an industrial scale.

### 4.6. Antimicrobial Activity of Bee Bread

Bee bread from different regions of Morocco was evaluated by Abouda et al. [83] for antibacterial activity against the following bacteria: *Staphylococcus aureus*, *Bacillus cereus*, *Pseudomonas aeruginosa,* and *Escherichia coli*. The results showed that all bee bread samples exhibited strong antimicrobial activities against the bacterial strains, with greater sensitivity to Gram-positive bacteria than Gram-negative bacteria.

Baltrušaitytė et al. [65] tested four samples of bee bread against *Staphylococcus aureus* and *Staphylococcus epidermidis*. The results obtained after the neutralization of the product and its treatment with catalase revealed that the samples of bee bread had non-peroxide antibacterial activity. Similarly, Ivanišov et al. [66] showed that samples of bee bread from five regions of Ukraine possessed antimicrobial activities against four bacterial strains, two Gram-positive—*Bacillus thuringiensis* and *Staphylococcus aureus*—and two Gram-negative—*Escherichia coli* and *Salmonella enterica*. The findings revealed that the minimum inhibitory concentrations varied between 6.40 µg mL^−1^ and 25.58 µg mL^−1^.

The hydromethanolic extract of Moroccan multifloral bee bread (35% *Anethum graveolens*, 24% *Quercus ilex*, 16% *Eucalyptus,* and 25% other pollens) was evaluated against six bacterial strains (*Bacillus cereus, Staphylococcus aureus, Escherichia coli, Enterobacter cloacae, Salmonella typhimurium,* and *Listeria monocytogenes*), and it was shown that the minimum inhibitory concentration (MIC) and minimum bactericidal concentration (MBC) values ranged between 0.04 mg/mL and 0.25 mg/mL and between 0.08 and 0.35 mg/mL, respectively. The bee bread sample was also effective against *Aspergillus fumigatus, Aspergillus ochraceus, Aspergillus niger, Penicillium funiculosum, Penicillium ochrochloron,* and *Penicillium verrucosum* var. *cyclopium*. The obtained MIC and MBC values ranged between 0.35 and 1 mg/mL and between 0.70 and 1.40 mg/mL, respectively [20] (Table 3).

In a study conducted by Didaras et al. [84], it was reported that the aqueous extract of Greek bee bread was effective against Gram-positive bacteria (MIC values ranging between 3.9 mg/mL and 48 mg/mL) and against Gram-negative bacteria (MIC values ranging between 7.8 mg/mL and 90.4 mg/mL). It has also been shown that bee bread ethanolic extracts (70%, 95%, 80%, and 50%) have strong antibiotic activity against Gram-positive bacteria (*Bacillus cereus, Clostridium perfringens, Staphylococcus aureus, Bacillus Subtilis,* methicillin-susceptible *Staphylococcus aureus, Staphylococcus* *epidermidis,* and *Listeria monocytogene*), Gram-negative bacteria (*Haemophilus influenza, Klebsiella pneumonia, Salmonella enteric, Escherichia coli, Shigella, Salmonella typhi*, and *Pseudomonas aeruginosa*)and fungi (*Candida albicans, Aspergillus niger*, *Candida glabrata, Candida tropicalis*, *Aspergillus clavatus, Aspergillus flavus, Aspergillus versicolor, Penicillium expansum, Penicillium chrysogenum,* and *Penicillium griseofulvum*) [52,85,86,87,88].

In most studies, it seems that Gram-positive bacteria are, in general, more susceptible to bee bread extract than Gram-negative bacteria. This may be due to the cytoplasmic membrane of Gram-negative bacteria, which contains less anionic phospholipids than Gram-positive bacteria; this particular composition is the cause of the resistance of certain Gram-negative bacteria to antibiotics [89].

Until now, the antiviral property of bee bread was revealed only in a study published by Didaras et al. [90] against the EV-D68 virus. The results showed promising IC_50_ values, ranging between 0.048 and 5.45 mg/mL, and CC_50_ values ranging between 0.17 and 8.60 mg/mL. Numerous reports have suggested that the antiviral effect of bee products is attributed to their content of polyphenols, such as caffeic acid, chrysin, galangin, and rutin [91,92]. Recently, in silico studies have shown that flavonoids may inhibit SARS-CoV-2 [93,94].

### 4.7. Proposed Mechanisms of Antimicrobial Action

The investigation of new, safer, and more effective antibacterial molecules from food and food by-products has attracted the interest of many researchers worldwide. Owing to their hydroxyl groups (OH), phenolic compounds and some antioxidant molecules interact with the bacterial cell membrane by hydrogen bonding and achieve their antibacterial action by two possible combined mechanisms. The first mechanismis through the disruption of the bacterial membrane structure and thus the loss of the cellular contents [95]. The second mechanism depends on the delocalization of electrons, which results in the depolarization of the cell membrane and the reduction in the pH gradient across the membrane, and thus a reduction in ATP synthesis [96]. The antibacterial efficacy of phenolic compounds is mainly influenced by the relative position of the hydroxyl group on the phenolic nucleus of each antioxidant molecule [95].

Bee bread contains different valuable molecules (Table 1) with potent bactericidal properties. Several studies have shown that flavonoids induce their antibacterial actions by targeting different pathways and mechanisms, including the inhibition of nucleic acid synthesis, the disruption of cytoplasmic membrane function, the modification of cell membrane permeability, and the interaction with some vital bacterial enzymes. A recent study conducted by Wang and coworkers demonstrated that quercetin, a ubiquitous flavonol, exerts its bactericidal effect on *Escherichia coli* and *Staphylococcusaureus*bydamaging the bacterial wall and membrane structure as well as by inhibiting bacterial protein synthesis [97]. Furthermore, quercetin inhibits bacterial energy metabolism and DNA synthesis [98]. It was shown that kaempferol, a flavonol compound commonly found in bee bread, inhibits DNA PriA helicase and decreases ATPase activity in *Staphylococcus aureus*, which suggests its application as a natural component in the development of new active antibiotics against *Staphylococcus aureus* [99]. The antibacterial effect of other flavonoids, such as apigenin, has also been evaluated. Yu et al. [100] reported that apigenin inhibits RNA polymerase and DNA gyrase in *Staphylococcus aureus*.

**Figure 2:**
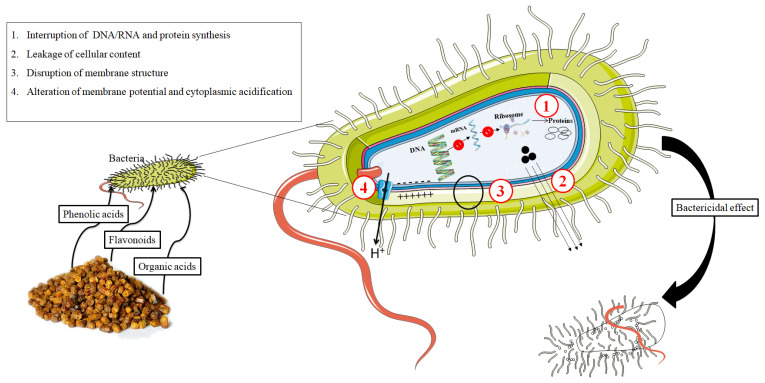
The possible antibacterial mechanisms of action of the bioactive molecules found in bee bread.

In addition to phenolic compounds, organic acids (aromatic and aliphatic) have been reported to possess bacteriostatic and bactericidal effects [101]. Organics acids can permeate facilely through the bacterial membrane; once inside the cells, they release protons (H^+^), decreasing intracellular pH, attacking macromolecules, and destabilizing bacterial walls [102]. The antibacterial properties of individual organic acids, such as gluconic, acetic, and formic acids, were widely investigated by several researchers. It was shown in one study that gluconic acid, a major organic acid present in bee bread, caused membrane cell depolarization and disrupted membrane integrity [103]. Moreover, a recent study showed that acetic acid is implicated in the sabotage of bacterial gene expression, including DNA replication enzymes, the elongation factors TU and GOS, polymerase alpha subunit, C-acetyltransferase 1OS, and chaperone proteins [104]. Besides its antibacterial effect, acetic acid exhibited a high inhibition of Conidia germination and aflatoxin production [105]. Finally, the antimicrobial efficacy of bee bread extracts is potentially due to the interaction between its active ingredients and microbial cells through one or more different mechanisms of action (Figure 2). Current data suggest that bee bread could be used as a promising antibiotic and natural food preservative ingredient.

**Table 3:** Antimicrobial and antiviral activities of bee bread.

Effect	Country	Type of the Study	Bacteria/Fungi/Virus Strains	Extract Used	Key Results	References
Antibacterial	Morocco	In vitro	Gram-positive bacteria: *Bacillus cereus* (food isolate), *Staphylococcus aureus* (ATCC 6538), and *Listeria monocytogenes* (NCTC 7973) Gram-negative bacteria: *Escherichia coli* (ATCC 35210), *Enterobacter cloacae* (human isolate), and *Salmonella typhimurium* (ATCC13311)	Hydromethanolic extract One gramof BB extracted twice with 30 mL of a mixture of methanol/water (80:20 *v/v*) for 60 min	Gram-positive bacteria: MIC values ranging between 0.4 and 0.175 mg/mL MBC values ranging between 0.08 and 0.35 mg/mL Gram-negative bacteria: MIC values ranging between 0.175 and 0.25 mg/mL MBC values were 0.35 mg/mL for all strains	[20]
Antifungal			*Aspergillus fumigatus* (ATCC 1022), *Aspergillus ochraceus* (ATCC 12066), *Aspergillus niger* (ATCC 6275), *Penicillium funiculosum* (ATCC 36839), *Penicillium ochrochloron* (ATCC 9112), and *Penicillium verrucosum* var. cyclopium (food isolate)		MIC values ranged between 0.35 mg/mL and 1 mg/mL, and MFC values ranged between 0.70 mg/mL and 1.40 mg/mL	
Antibacterial	Morocco	In vitro	Gram-positive bacteria: *Staphylococcus aureus*, three strains of *Streptococcus*, *Bacillus cereus* Gram-negative bacteria: Three strains of **Escherichia coli*, *Salmonella enteritidis*, three strains of *Pseudomonas aeruginosa**.	DMSO (10%) 50%(*v/v*)	Gram-positive bacteria: The inhibition diameters ranged between 9 and 27 mm Gram-negative bacteria: The inhibition diameters ranged between 9 and 29 mm	[83]
Antibacterial	Lithuania	In vitro	Gram-positive bacteria: *B. cereus* 1801, methicillin-resistant *Staphylococcus aureus* (MRSA) M87fox, *E. faecalis* 86, S. epidermidis, *S. haemolyticus*, and *Streptococcus mutans* Gram-negative bacteria: *A. baumannii*17-380, *C. freundii, E. cloacae, Enterococcus faecium* 103, *Klebsiella pneumoniae,P. multocida, P. mirabilis, Pseudomonas aeruginosa* 17-331, and *Salmonella enterica* 24 SPn06.	One gram of bee products was dissolved in 20 mL of aqueous ethanol (500 mL/L) for 6 h	The inhibition diameters ranged between 9 and 22.8 mm	[73]
	Greek	In vitro	Gram-positive bacteria: Methicillin-resistant *Staphylococcus aureus* strain 1552 Gram-negative bacteria: Carbapenem-resistant *Pseudomonas aeruginosa* strain 1773, *Salmonella typhimurium*, and *Klebsiella pneumonia*	Different amounts of bee bread were extracted with 10 mL of water at room temperature for 24	Gram-positive bacteria: MIC values ranging between 3.9 mg/mL and 48 mg/mL MBC values ranging between 3.9 mg/mL and 48 mg/mL Gram-negative bacteria: MIC values ranging between 7.8 mg/mL and 90.4 mg/mL MBC values ranging between 9.9 mg/mL and >93.2 mg/mL	[84]
	Poland	In vitro	Gram-positive bacteria: *S. aureus*ATCC 25923, *S. aureus* ATCC 29213, *S. epidermidis*ATCC 12228, six MSSA (methicillin-susceptible *Staphylococcus aureus*), and three MRSA (methicillin *resistant Staphylococcus aureus*) Gram-negative bacteria: *P. aeruginosa* ATCC 27853 and *E. coli* ATCC 25922	BB extracted in 70% ethanol at *v/w* ratio of 7:1 at ambient temperature for 2 h	Gram-positive bacteria: MIC values ranging between 2.5 and 10% (*v/w*). MBC values ranging between 2.5 and 20% Gram-negative bacteria: MIC values ranging between 5 and 20% (*v/w*) MBC values ranging between 10 and >20% (*v/w*)	[85]
Antibacterial	Ukraine	In vitro	Gram-negative bacteria: *Escherichia coli* CCM 3988 andSalmonella enterica subs. *Enterica* CCM 3807 Gram-positive bacteria: *Bacillus thuringiensis* CCM 19 and *Staphylococcus aureus* subs. *aureus* CCM 4223.	BB was extracted with ethanol; 0.1 g of BB was extracted with 20 mL of ethanol (80%) for 2 h	Gram-positive bacteria: MICvalues ranging between 12.81 and 27.20 μg/mL Gram-negative bacteria: MIC values ranging between 6.40 and 13.64 μg/mL	[66]
Antiviral	Greece	Cell culture method	Enterovirus D68	BB was dissolved in Dulbecco’s Modified Eagle cell culture medium for one hour at room temperature	IC_50_ ranged between 0.048 and 5.45 mg/mL CC_50_ ranged between 0.17 and 8.60 mg/mL	[90]
Antibacterial	Malaysia	In vitro	Gram-positive bacteria: *L.monocytogene, S. aureus*,and *B. cereus* Gram-negative bacteria: *E.coli, Salmonella*, and *P. aeruginosa*	Twenty grams of BB were extracted twice with 80 g of 95% (*v/v*) ethanol for 12 h and then with 40g of 95% (*v/v*) ethanol.	Gram-positive bacteria: The inhibition diameters ranged between 0 and 284.44 mm Gram-negative bacteria: The inhibition diameters ranged between 0 and 312.22 mm	[86]
Antibacterial	Malaysia	In vitro	Gram-negative bacteria: *Klebsilla pneumonia*, *Escherichia coli*, *Shigella*, and *Salmonella typhi*	Fifty grams of BB were extracted with 500 mL of 70% ethanol and with water and hot water for 72 h at room temperature	*Shigella* (MIC: 1.617 µg/mL), followed by *Salmonella typhi* (MIC: 1.813 µg/mL), *E. coli* (MIC: 1.914 µg/mL), and *Klebsilla pneumonia* (MIC: 1.923 µg/mL)	[87]
Antibacterial	Egypt	In vitro	Gram-positive bacteria: *Staphylococcus aureus* (ATCC 6538) and *Bacillus Subtilis*(ATCC 6633) Gram-negative bacteria: *Escherichia coli* (ATCC 8739) and *K. pneumonia* (ATCC 13883)	One hundred grams of BB were soaked in 200 mL of ethanol 80% for 24 h and then homogenized for 30 min	Gram-positive bacteria: The inhibition diameters ranged between 24 and 26 mm Gram-negative bacteria: The inhibition diameters ranged between 12 and 18 mm	[52]
Antifungal			Unicellular fungi *Candida albicans* (ATCC 10221) and filamentous fungi *Asp. niger*		The inhibition diameter for *Candida albicans* was 15 ± 0.73 mm No effect against *Aspergillus niger*	
Antibacterial	Ukraine	In vitro	Gram-positive bacteria: *Bacillus cereus* CCM 2010, *Clostridium perfringens* CCM 4435, and *Staphylococcus aureus* subsp. *aureus* CCM 4223 Gram-negative bacteria: *Haemophilus influenza* CCM 4456, *Klebsiella pneumoniae* CCM 2318, and *Salmonella enterica subsp. enterica* CCM 3807	BB was extracted by maceration using 50% ethanol	Gram-positive bacteria: The inhibition diameters ranged between 9 and 16 mm Gram-negative bacteria: The inhibition diameters ranged between 1.7 and 5 mm	[88]
Antifungal			*Candida albicans* CCM 8186, *Candida glabrata* CCM 8270, *Candida tropicalis* CCM 8223, *Aspergillus clavatus, Aspergillus flavus, Aspergillus versicolor, Penicillium expansum, Penicillium chrysogenum*, and *Penicillium griseofulvum*		Candia species: The inhibition diameters ranged between 4 and 8 mm Fungi species: The inhibition diameters ranged between 1 and 3.7 mm	

## 5. The Pharmacological Effect of Bee Bread: In Vivo Investigations

Moroccan bee bread was evaluated in type 1 diabetic Wistar rats; the sample was extracted using ethyl acetate and it was given orally at the dose of 100 mg/kg for 15 days. The results showed that the bee bread was effective at decreasing blood glucose levels, it protected against loss weight induced by diabetes, it possessed a hypolipidemic effect, and it protected diabetic rats against the increase in the coronary risk index, atherogenic index, and cardiovascular index [64]. In addition, the ethanolic extract of the same sample was evaluated by Bakour et al. [51] against the toxicity of titanium dioxide nanoparticles in Wistar rats. The results revealed that the bee bread reduced the elevation of aspartate aminotransferase (AST), alanine aminotransferase (ALT), lactate dehydrogenase (LDH), blood glucose, sodium, potassium, chloride, total cholesterol (TC), and low-density lipoprotein cholesterol (LDL), and it exhibited a highly protective effect against the decrease in albumin and total protein. Additionally, bee bread, at a dose of 500 and 750 mg/kg, alleviated the biochemical changes induced by aluminum in rats by increasing hematocrit, hemoglobin, red blood cells, mean corpuscular hemoglobin (MCH), mean corpuscular volume (MCV), mean corpuscular hemoglobin concentration (MCHC), urine sodium, and creatinine clearance and decreasing platelets, monocytes, lymphocytes, leukocytes, ALT, AST, C-reactive protein (CRP), and blood urea [63] (Table 4).

Malaysian bee bread was tested in two studies in high-fat diet-induced obese rats; the dose used in both investigations was 0.5 g/kg via oral routes using distilled water. The first study conducted by Eleazu et al. [30] revealed that the administration of bee bread in obese rats decreased the percentage change in body weight, BMI index, kidney weight, MDA concentrations, inflammatory cells in kidney tissue, NFkB, TNF-α, interleukin-l-beta, and Bax, while it increased the levels of SOD, GPx, GST, and TAA and reduced the Bowman’s capsule space in the urinary chambers of the kidneys. In the second study conducted by Othman et al. [106], the administration of bee bread in obese male Sprague–Dawley rats improved their lipid profile, aortic inflammatory markers, and their impaired vasorelaxation activity, and showed its ability to enhance nitric oxide release and promote endothelial nitric oxide synthase (eNOS) and cyclic guanosine monophosphate (cGMP) immunoexpression. The same protocol forhigh-fat diet-induced obese rats was used by Suleiman et al. [107] to examine the protective effect of bee bread at a dose of 0.5 g/kg once daily for 12 weeks on testicular oxidative stress, inflammation, apoptosis, and lactate transport in the testes of obese rats (Table 4).

Several studies have examined the ability of bee bread to lower blood pressure. These studies have evaluated the effects of the oral administration of bee bread extract on vascular inflammation and impaired vasorelaxation in vivo using an obesity-induced vascular damage rat model [30,108]. The authors reported that bee bread improved the impaired vasorelaxation activity by enhancing the release of nitric oxidase, endothelial nitric oxide (sNOS), and cyclic guanosine monophosphate immunoexpression, which provide vascular protection. In addition, it has been proven that bee bread boosts the aortic antioxidant activities impaired in high-fat diet-induced obese rats by ameliorating aortic proinflammatory markers (tumor necrosis factor-α and nuclear factor—κβ) and preventing aortic structural damage perturbation in the vasorelaxation response to acetylcholine [30]. The phytochemical analysis of bee bread reveals its diverse phytochemical compounds with high antioxidant potential, including ferulic acid, caffeic acid, kaempferol, apigenin, and isorhamnetin. These bioactive molecules have proven their ability to act as antiatherogenic agents against the perturbations induced by a high-fat diet [108].

In the same context, Martiniakova et al. [109] reported that the oral administration of monofloral bee bread extract (*Brassica napus* L.) significantly lowered blood glucose levels, prevented lipid abnormalities, and impaired the bone morphology of Zucker diabetic fatty rats. In addition, Haščík et al. [110] showed the effect of dietary bee bread powder on the chemical composition of Japanese quails meat. In the breast muscle of quails, the bee bread decreased the water content, increased the crude protein content, and decreased fat and cholesterol, while in the thigh muscle of quails, the dietary bee bread increased the water content, fat, and cholesterol.

In Turkey, the effect of bee bread on leptin and ghrelin expression in obese rats was studied by Doğanyiğit et al. [111] in Sprague–Dawley adult female rats using two doses via oral administration; the results showed that bee bread decreased ghrelin immunoreactivity, increased leptin immunoreactivity, decreased the apoptotic cell numbers in the hypothalamus, and decreased MDA levels. In addition, it was shown that bee bread from Slovakia reduced femoral bone structure and improved glucose and lipid metabolism in Zucker diabetic fatty rats [112]; bee bread from China has also been shown to have a capacity for the regulation of lipid metabolism [113].

**Table 4:** Pharmacological properties of bee bread in vivo.

Country	Functional Effect	Protocol Used	Palynological Analysis of Bee Bread (BB)	The Majority of Compounds Identified	Extraction Used	Concentration/ Treatment Duration	Administration Routes	Model Used	Results Obtained	References
Morocco	Hypoglycemic, hypolipidemic, and hepatoprotective effects	Streptozotocin-induced diabetic rats	*Bupleurum spinosum, Anethum graveolens, Calendula officinalis, Anacyclus, Quercus ilex, Eucalyptus, Punica granatum,* and *Acacia*	Isorhamnetin-*O*-hexosyl-*O*-rutinoside was the major phenolic compound present, in addition to tocopherols	Ethyl acetate extract	100 mg/kg For 15 days	Oral	Wistar rats	↓ Blood glucose levels Protect against weightloss ↓ TC, ↑ HDL, ↓ TG, ↓ LDL, ↓ VLDL Coronary risk index ↓ Atherogenic index ↓ Cardiovascular index	[64]
Morocco	Protective effects against anemia, inflammation, and hepato-renal toxicity	Toxicity of aluminum	One sample (not determined)	Polyphenol content: 14.88 ± 0.98 mg GAE/g Flavonoid content: 1.67 ± 0.12mg QE/g	Maceration for one week in 70% ethanol under agitation	500 and 750 mg/kg	Oral	Male Wistar rats	↑ Hematocrit, hemoglobin, red blood cells, MCH, MCV, and MCHC ↓ Platelets, monocytes, lymphocytes, and leukocytes. ↓ ALT, AST, and CRP ↓ Blood urea ↑ Urine sodium and creatinine clearance	[63]
Morocco	Protective effects against the toxicity induced by titanium dioxide in biochemical parameters of the brain, liver, and kidney tissues	Titanium dioxide nanoparticles induced toxicity in rats	*Bupleurum spinosum, Anethum graveolens, Calendula officinalis, Anacyclus, Quercus ilex, Eucalyptus, Punica granatum,* and *Acacia*	Isorhamnetin-*O*-hexosyl-*O*-rutinoside was the major phenolic compound present, in addition to tocopherols	Ethanolic extract	100 mg/kg For 30 days	Oral	Wistar rats	↓ AST, ↓ ALT, ↓ LDH ↓ TC, ↑ HDL, ↓ LDL ↓ Blood glucose levels ↓ Urea, ↑ Albumin, ↑ Total protein ↓ Sodium, ↓ potassium, ↓ chloride Protection against histopathological changes in the brain, kidneys, and liver	[51]
Slovakia	Alleviates lipid abnormalities and impaired bone morphology in obese Zucker diabetic rats	Obese Zucker diabetic rats	Monofloral (*Brassica napus* L.)	Vitamin A, vitamin E vitamin C, vitamin B2, vitamin B3, and Beta carotene	Mixed with distilled water	500 and 700 mg/kg	Oral	Zucker diabetic Fatty rats	↓ Blood glucose level ↓ TC, ↓ triglycerides ↑ the relative volume of trabecular bone ↑ trabecular thickness, enhanced density of secondary osteons, accelerated periosteal bone apposition, and improved blood flow	[109]
Malaysia	Protective effect against testicular oxidative stress, inflammation, apoptosis, and lactate transport in the testes of obese rats	High-fat diet (HFD)	One sample (not determined)	Riboflavin, thiamine, vitamin A, vitamin E, Fe, Cu, Zn, apigenin, caffeic acid, ferulic acid, isorhamnetin, andkaempferol	Blended and used as a fine powder	0.5 g/kg/day) once daily for 12 weeks.	Oral	Male Sprague–Dawley rats	↓ Final body weight and weight gain ↓ Lee obesity index, BMI, and energy intake. ↓ TC, ↓ TG, ↓ LDL, ↑ HDL ↓ The absolute and relative weights of the epididymal fat. Histological improvements of the testis. ↑ the activities of SOD, CAT, GPx, GST, GR, TAC ↓ the level of TBARS. ↑ The mRNA expression of Nrf2, SOD, CAT, and GPx. ↓ The mRNA transcript levels of pro-inflammatory *Nf-κb*, Tnf-*α, iNos*, and *Il-1β* genes. ↓ the activities and mRNA expression pro-apoptotic (p53, Bax, Bax/Bcl2, Caspase-8, Caspase-9, and Caspase-3) genes in the testes ↑ the mRNA levels of glucose transporters (Glut1 and Glut3), monocarboxylate transporters (Mct2 and Mct4), and lactate dehydrogenase type C (Ldhc) ↑ lactate utilization. ↑ PCNA immunoexpression	[107]
Turkey	The effects of bee bread on leptin and ghrelin expression in obese rats	Female rats became obese with a high-fat diet	One sample (not determined)	Protein: 13.56g/100g Carbohydrate: 30.60 g/100g Dietary fiber: 18.18g/100g Fat: 21.69g/100g	Distilled water	Concentration1: 100 mg/kg/day Concentration2: 200 mg/kg/day	Oral	Sprague–Dawley adult female rats	↓ Ghrelin immunoreactivity in obese rats that received BB (100 mg/kg/day and 200 mg/kg/day). ↑ Leptin immunoreactivity in obese rats that received BB (100mg/kg/day and 200 mg/kg/day). ↓ Apoptotic cell numbers in hypothalamus tissue in obese rats that received BB (100 mg/kg/day and 200 mg/kg/day) ↑ SOD activity in obese rats that received BB (200 mg/kg/day). ↓ Ghrelin amounts in obese rats that received BB (200 mg/kg/day). ↑ Leptin amounts in obese rats that received BB (200 mg/kg/day). ↓ MDA levels in obese rats that received BB (200 mg/kg/day)	[112]
Slovakia	The effects of dietary bee bread powder on the chemical composition of quail meat.	Dietary supplementation of bee bread powder in quails	One sample (not determined)	(not determined)	Bee bread powder	2 g or 4 g or 6 g per 1 kg of feed mixture	Oral	Japanese quails	The effect of BB supplementation on the chemical composition of breast muscle in quails: ↓ Water content ↑ Crude protein ↓ Fat ↓ Cholesterol The effect of BB supplementation on the chemical composition of thigh muscle in quails ↑ Water content ↑ Fat ↑ cholesterol	[110]
China	Regulation of lipid metabolism	SPF rats	Not mentioned	-		80, 400, and 800 mg/kg during 20 days	Oral	Rats	↓ Fatty acid Synthase ↓ Acetyl CoA carboxylase ↓ lipoprotein lipase ↓ Total cholesterol level, ↓ triglycerides, ↑ HDL, ↓ LDL	[111]
Malaysia	Bee bread attenuates high-fat diet (HFD) induced renal pathology in obese rats via The modulation of oxidative stress, down regulation of NF-kB mediated inflammation, and Bax signaling	High-fat diet	One sample (not determined)	Protein: 3.37 ± 0.30% Lipid: 4.32 ± 0.17% Carbohydrate: 82.45 ± 0.36% Iron:0.02 ppm Copper: 0.01 ppm Zinc: 0.002ppm Vitamin A: 146.8 ± 6.21 (mg/100 g) Vitamin E: 46.27 ± 0.67 (mg/100 g) Thiamine: 6.20 ± 0.06 (mg/100 g) Riboflavin: 0.50 ± 0.00 (mg/100 g)	Distilled water	0.5g/kg	Oral	Male Sprague–Dawley rats	↓ % change in body weight, BMI index in rats that received HFD treated or protected with BB. ↓ Kidney weight in rats that received HFD treated or protected with BB. ↑ SOD, GPx, GST, TAA in rats that received HFD treated or protected with BB ↓ MDA concentrations in rats that received HFD treated or protected with BB ↓ Inflammatory cells in kidney tissues of HFD groups treated or protected with BB. ↓ Bowman’s capsule space in the urinary chamber of kidneys of HFD groups treated or protected with BB. ↓ NFkB, TNF-α, interleukin-1-beta, and Bax in rats that received HFD treated or protected with BB	[30]
Malaysia	Bee bread ameliorates the impaired vasorelaxation response to ACh by improving the eNOS/NO/cGMP-signaling pathway in obese rats	High-fat diet	One sample (not determined)	Potassium (7323.04 mg/kg), magnesium (1530.87 mg/kg), calcium (1108.48 mg/kg), sodium (252.73 mg/kg) iron (56.58 mg/kg), zinc (42.36 mg/kg), copper (11.05 mg/kg), and selenium (0.13 mg/kg)	Distilled water	0.5 g/kg	Oral	Male Sprague–Dawley rats	BB improves the lipid profile, aortic inflammatory markers, and impaired vasorelaxation activity. BB enhances nitric oxide release, promotes endothelial nitric oxide synthase (eNOS) and cyclic guanosine monophosphate (cGMP) immunoexpression.	[106]
Slovakia	Reduced femoral bone structure and improved glucose and lipid metabolism in Zucker diabetic fatty (ZDF) rats	Obese Zucker diabetic rats	Not mentioned	-	The sample was crushed and mixed with distilled water	500 mg/kg	Oral	Diabetic fatty rats	↓ Fasting blood glucose level ↓ Total cholesterol level, ↓ triglycerides, protection against body weight loss, ↓ ALP activity, ↓ cortical bone surface, relative bone volume, ↑ trabecular bone, ↑ trabecular thickness, and trabecular bone surface	[113]

↑: Increase; ↓ Decrease.

## 6. The Use of Bee Bread in Clinical Studies

### 6.1. Hepatoprotective Effect

Bee bread was tested by Čeksterytė et al. [114] in patients with chronic hepatitis. The most important clinically relevant finding was a significant improvement in blood parameters, including erythrocyte count, hemoglobin, leukocytes, C-reactive protein (CRP), blood sugar, aspartate aminotransferase (AST), alanine aminotransferase (ALT), and bilirubin.

### 6.2. Anti-Atherogenic Dyslipidemic Effect

Kas’ianenko et al. [115] evaluated the effectiveness of treating patients with atherogenic dyslipidemia with a mixture of honey, pollen, and bee bread. The parameters of atherogenic dyslipidemia were examined in 157 patients (64 men and 93 women) aged 39 to 72 years. These patients were divided into four groups: (1) treated with a lipid-lowering diet only; (2) treated with a lipid-lowering diet and honey or pollen; (3) treated with bee bread only; and (4) treated with honey and pollen. The results obtained showed that a significant lipid-lowering effect was recorded in the patients taking honey in combination with pollen (total cholesterol decreased by 18.3% and LDL-C decreased by 23.9%) and bee bread (total cholesterol decreased by 15.7% and LDL-C decreased by 20.5%).

### 6.3. Strengthening Visual Acuity

Jarušaitienė et al. [116] studied refractive status, visual acuity, and ocular prophylaxis in 34 children (aged 6 to 17) with thyroid disease before and after taking bee bread. The results of this study showed an increase in visual acuity in the subjects who used bee bread.

### 6.4. Ergogenic Effect and Improvement of Athletic Performance

Chen et al. [117] examined the effects of bee bread supplementation during recovery on athletic performance. Twelve athletes were chosen for the study. During the experimental trials, the participants ran on a treadmill for 90 min and then rested for four hours. During this recovery period, the participants consumed 30 g/h of bee bread or a placebo. Heart rate and tympanic temperature were measured at 20-min intervals during this period. Blood samples were taken to determine plasma glucose, hemoglobin, and hematocrit. Participants then performed a 20-min trial on a treadmill. The distance traveled in the bee bread trial was significantly longer than the placebo trial (3.45 ± 0.4 km vs. 3.24 ± 0.4 km, respectively). The plasma glucose levels in the bee bread trial were significantly higher compared to those in the placebo trial during recovery. These results showed that the supplementation of bee bread during the recovery phase appears to provide improved athletic performance in athletes.

In a second study conducted by Fadzel et al. [118], bee bread supplementation was tested on the running performance of athletes. This time, the athletes were given 20 g of bee bread or a placebo every day for 8 weeks. After the first experimental run, there was a 4-week washout period. Then, they continued with the supplementation for 8 weeks before the second experimental trial. During the experimental trials, the participants ran at 60% VO_2_ max for 90 min, immediately followed by a 20-min trial. Heart rate, oxygen uptake, ear temperature, perceived exertion rate, ambient temperature, and relative humidity were recorded during the tests. Blood tests were performed to determine the levels of plasma glucose and free fatty acids. The results of the study showed that there was no significant difference between the bee bread test and the placebo test for heart rate, oxygen uptake, tympanic temperature, the rate of perceived exertion, and plasma glucose levels. However, the distance traveled in the bee bread trial was significantly greater than that in the placebo trial (3.41 ± 0.2 km vs. 3.28 ± 0.2 km, respectively). In addition, the free fatty acid plasma levels in the bee bread trial were significantly higher than in the placebo trial. This allowed the researchers to conclude that supplementation with bee bread appeared to improve the running performance of athletes [118].

## 7. Bee Bread Adulteration

Hive products, namely honey and bee pollen, have long since been considered as a functional food for their broad-based composition and wide range of biological activities. Recently, the use of these products has increased all over the world because of public awareness of their nutritional and therapeutic properties, which could lead to a substantial economic-motivated adulteration.

Bee bread is a bee-derived product with an extended range of bioactive compounds; however, it is still not well-known and has a scarce production in the apiaries because of the difficult harvesting methods and the beekeepers’ conviction that bee bread reserves in the hives should not be reduced to preserve the colonies’ development [119]. The growing consumer attention regarding the health benefits of bee bread has increased its demand in the market compared with its production, which sets the stage for its adulteration and becomes an issue of concern for all bee product producers. However, the international scientific community has just started to define the regulation and quality criteria of bee products. Thus far, there are no acknowledged standards for bee bread, which makes it difficult to detect its falsification [120,121].

Based on the composition previously discussed in the present work, the detection of fraudulent bee bread manipulations requires the combination of multiple techniques mainly based on the composition of pollen in bee bread [122], including the identification of the floral origin of the bee pollen (starter matrices to the bee bread production) by a palynological survey [123] and the spectroscopic determination of the chemical composition of the bee bread, notably its vitamins (vitamin C, vitamin E, and β-carotene), amino acids, and fatty acids (taking into consideration the relationship between the botanical origin and chemical composition of bee pollen) [124,125]. Recently, advanced foodomics technologies have been used to characterize food products and have been applied to bee bread and other bee products to assess authenticity, safety, and quality issues and to determine the bioactive compounds present and their biological activities. In their review article, Kafantaris et al. [126] summarized the different methods used for the study of bee bread, including genomics as an alternative for the palynologic/microscopic determination of the botanical origin using the metabarcoding of DNA isolated from bee pollen and bee bread samples. The proteomics and metabolomics technologies can be performed using GC-MS, HPLC-DAD, SDS-PAGE, 2D-electrophoresis, and MALDI–MS methods for the identification of proteins, enzymes, fatty acids, the phenolic profile, and carotenoid composition. Additionally, metagenomics can beapplied to investigate the microbial community of bee bread, especially lactobacillus bacteria [126].

On this basis, we can conclude that the determination of the botanical origin of bee pollen, its chemical and enzymatic composition, and the characterization of its microbiome and biological properties are necessary for the authenticity, traceability, and safety of bee bread.

## 8. Conclusions

In conclusion, bee bread is a functional food produced by the fermentation of bee pollen and honey in the honeycombs of the hives. It contains a wide range of components, such as sugars, polyphenols, vitamins, and free amino acids. Bee bread is characterized by considerable pharmacological properties, proven in vitro and in vivo. Thus, it can be stated that bee bread offers a wide field for the exploitation of its benefits in the food and pharmaceutical industries.

## Data Availability

Data are available upon request.
